# The Impact of Social Media Videos on Quantitative Health Outcomes: Systematic Review

**DOI:** 10.2196/77578

**Published:** 2026-02-19

**Authors:** Fangyue Chen, Tricia Tay, Hannah Thould, Chatchaya Nangsue, Simon Dryden, Amish Acharya, Ara Darzi, Kate Grailey

**Affiliations:** 1 Fleming Initiative, Institute of Global Health Innovation, Imperial College London, London, SW7 2AZ, United Kingdom, 44 07510888677; 2 Helix Centre, Institute of Global Health Innovation, Imperial College London, London, United Kingdom; 3 University Hospitals Bristol and Weston NHS Foundation Trust, Bristol, United Kingdom; 4 Faculty of Medicine, Chiang Mai University, Chiang Mai, Thailand

**Keywords:** social media, online video, health outcome, PRISMA, Preferred Reporting Items for Systematic Reviews and Meta-Analyses

## Abstract

**Background:**

Social media has transformed the landscape of health communication. Video content can optimally activate our cognitive systems, enhance learning, and deliver accessible information. Evidence has suggested the positive impact of videos on health knowledge and health-related behaviors, yet the impact of social media videos on quantitative health outcomes is underresearched. Evaluating such outcomes poses unique challenges in measuring exposure and outcomes within internet-based populations.

**Objective:**

We aimed to evaluate the impact of social media videos on quantitative health outcomes, examine methodologies used to measure these effects, and describe the characteristics of video interventions and their delivery.

**Methods:**

In accordance with PRISMA (Preferred Reporting Items for Systematic Reviews and Meta-Analyses) guidelines, MEDLINE, Embase, Web of Science, CINAHL, and Google Scholar were searched. Studies were eligible if they were original research evaluating long-form social media video interventions addressing any health-related condition, delivered via social media platforms, and reported quantitative health outcomes. The primary outcome was the effect of social media videos on quantitative health outcomes. Additional outcomes included participant characteristics, video features, delivery methods, and the use of theoretical frameworks. A narrative synthesis was conducted. A subgroup meta-analysis was performed to synthesize health outcomes mentioned in 2 or more studies with sufficient homogeneity. Risk of bias assessment was conducted using Cochrane Risk of Bias 2, ROBINS-I, or National Institutes of Health Quality Assessment Tool, depending on the study design. One reviewer screened titles and abstracts. Two reviewers independently conducted full-text screening, data extraction, and risk of bias assessment.

**Results:**

A systematic search was conducted on October 25, 2023, and was updated on June 12, 2025, yielding a total of 41,172 records after duplicate removal. Sixteen studies were included, involving 4158 participants. Mental health–related conditions were the most studied (10 studies). Most video interventions were delivered via YouTube (12 studies). Studies have reported that video interventions were associated with significant improvements in peri-procedural anxiety, mood, and physical activity levels, although most findings were limited to individual studies with variable methodological quality. Three studies that developed videos with user input and theoretical frameworks significantly impacted study-specific primary outcomes. A subgroup meta-analysis demonstrated a significant moderate impact of online video interventions in improving peri-procedural anxiety (standard mean difference=0.57, 95% CI 0.09‐1.05). All but one study showed some concern or high risk of bias.

**Conclusions:**

We demonstrated a potential positive impact of social media videos on quantitative health outcomes, notably in improving peri-procedural anxiety. Videos developed with user input and theoretical frameworks significantly impacted study-specific primary outcomes. Nevertheless, there is the need to shift focus toward measuring physical health–related outcomes and to develop better designed, innovative methodologies to measure the impact that can better simulate the social media environment.

## Introduction

Social media can be defined as “websites and computer programs that allow individuals to communicate and share information, opinions, pictures, videos, and other formats on the internet” [1]. Its growing presence within the health care context has facilitated communication between the public, patients, and health care professionals, as exemplified by its usage during the COVID-19 pandemic for organizations to disseminate information to the public [2,3] and for providing peer support spaces for patients suffering from a range of conditions, such as cancer and mental health illnesses [4,5].

One form of social media communication is through online videos, such as those hosted on YouTube, the largest video–based platform with 2.5 billion monthly active users globally [6]. Videos can simultaneously present complementary visual and auditory information, which optimally activate our cognitive systems and lead to better learning, as shown by previous meta-analyses of the effectiveness of learning with multimedia [7,8]. In addition, videos ensure consistency in the delivery of educational content and can be designed to cater to individuals with low literacy [9]. Viewers watching the videos may have a perceived greater autonomy over learning, as they can control the progress of the video [10], while video creators can incorporate video editing processes to improve learning experience [10,11].

Social media videos differ from traditional formats by their potential for a wide reach and the added level of interactivity from predominantly 1-way to 2-way forms of communication [12,13]. Within various health care disciplines, such videos have been developed to tackle a range of issues, including improving community health literacy, facilitating community building by patients sharing their illness journey [14], communicating research [15], and medical education [2]. An example of a popular health-related social media video includes a video of antibiotic pills singing “keep antibiotics working,” released as part of an antimicrobial resistance awareness campaign by UK Health Security Agency in 2017 [16]. While the main intention of the campaign was to garner online pledges from individuals to agree to undertake actions relating to antibiotic usage on the dedicated campaign website, the number of views from the video—over 1.3 million times to date—has exceeded the number of pledges the campaign has so far gained, and social media has directed the greatest level of traffic to the campaign website [9,16,17].

Despite the potential of health-related social media content, including those delivered through video formats, in impacting individuals’ health, as well as how they interact with health care services globally, how such impact translates to changes in individuals’ health-related outcomes remains under-researched. Health outcomes can be defined by health impacts resulting from a condition, event, or intervention [18]. These can be measured clinically (blood pressure, laboratory testing), self-reported (eg, quality of life measures), or observed (eg, changes in gait) [19]. Discerning such impact will enable more tailored governance, adaptation, and integration of such tools, so as to better understand and harness the value of social media within health care systems [20,21].

Previous systematic reviews have examined the impact of health-related videos with limited inclusion of and emphasis on the rise of social media videos. They have largely assessed the effectiveness of video interventions in terms of improvement in learning outcomes, such as knowledge and skills, and their impact on health-related behaviors, such as attendance to disease screening and lifestyle modifications (eg, smoking cessation) [10,22]. The impact of health-related social media videos beyond the openly available engagement analytics is relatively under-researched [23]. To our knowledge, no systematic review has collectively examined the impact of social media videos on quantitative health outcomes. Challenges in conducting trials to test the impact of online interventions include the difficulties in identifying an appropriate control, ensuring the control group has no access to the intervention on a publicly available platform, and collecting measurable outcomes from an audience who accesses content over the internet [24,25].

This systematic review aims to explore and synthesize evidence on the impact of social media online videos on quantitative health outcomes. We began by synthesizing the methodology used to evaluate the measurable health impact of online social media videos and in turn explored whether there was evidence that such videos could have an impact on quantitative health outcomes.

## Methods

This systematic review was conducted in accordance with the PRISMA (Preferred Reporting Items for Systematic Reviews and Meta-Analyses) guidelines (Checklist 1)[26]. The study protocol was registered on PROSPERO (CRD42023474648).

### Eligibility Criteria

Studies were eligible for inclusion if they reported the use of an online video intervention, defined as “pre-recorded multimedia that combine moving images and audio” found on a publicly available, freely accessible online social media platform that allows online communication between users, and discussed any health-related conditions, targeting any individuals with or without health conditions [10]. Currently, there are 2 formats of videos on social media—the traditional long-form videos and “Shorts,” the latter defined by vertical videos that are less than 180 seconds in duration and are presented to users passively and sequentially, with which users can choose to watch or swipe away [27]. While “Shorts” have gained global popularity, their brief, varied, and uncorrelated features have been associated with the development of addiction and impaired attention span, thus negatively impacting users [28,29]. Given different ways in which audience interact with long-form or “Shorts” videos, which can translate to the heterogeneous mechanisms in which different video forms can impact audience’s health-related behaviors and outcomes, this systematic review only included studies that described videos in long-form. In addition, if a study described several interventions, the online video would need to be the main intervention, rather than a part of the intervention package. To illustrate, studies would be included if they described a video intervention that was sent to participants through an SMS text, or if they described a video that included additional materials that were collectively presented onto the social media platform (eg, linked within the video description section). If the video and other information formats were delivered separately, so that it would be difficult to discern whether the study outcomes resulted from the video alone, the study would not be eligible. Studies were eligible if they measured quantitative health outcome or outcomes, including condition-specific outcomes, such as mini-mental status examination in dementia, and more general health outcomes, such as quantitative measures of quality of life [30,31]. Self-reported outcome measures using validated reporting tools were included. Studies with or without a comparator or comparators were included. There were no restrictions on the publication year and language. Literature published in Chinese was translated by a native Chinese speaker. All other languages were translated by Google Translate [32].

Studies were included if they were original research studies including randomized controlled studies, pre-post study design, nonrandomized controlled trials (RCTs), and surveys regarding the impact of the intervention. Review papers, such as narrative reviews, overviews, systematic reviews, and meta-analyses, and informal publication types, such as case studies, commentaries, letters to the editor, editorials, meeting abstracts, and proceeding papers, were excluded given the focus on primary research and the need for full methodological details.

### Information Sources

The following databases were searched: MEDLINE OVID (1946 to date of search), Embase Ovid (1947 to date of search), Web of Science Core Collection (1970 to date of search), and CINAHL EMBSCO (1981 to date of search). In addition, Google Scholar was searched and ranked by relevance. The first 1000 results of this search were reviewed, in line with Google Scholar’s capabilities [33].

### Search Strategy

Search terms were developed with a librarian experienced in conducting systematic searches. Using the PICO (population, intervention, comparator, outcome) strategy, population was humans of any age groups; intervention was online video as defined in the eligibility criteria; comparator included no intervention, standard intervention, or any other interventions that can impact health outcomes; outcome included any quantitative health–related outcomes. Two main search terms “online video” and “health” were used, which best reflected the PICO strategy. In addition, major online social media video platforms that hosted predominantly long-form videos were included as search terms in place of “online video,” including “YouTube,” “Vimeo,” “Dailymotion,” and “Facebook Watch.” The full search strategy can be found in Multimedia Appendix 1.

### Selection Process

All papers from the systematic search were imported into Covidence, a systematic review reference management system [34]. Screening took place in 2 stages. One reviewer (FC) performed abstract and title screening, selecting papers that investigated online videos and had mentioned the measure of quantitative health–related outcomes as defined in the eligibility criteria above. If it was unclear whether the outcome or outcomes met the eligibility criteria, the papers would be included for full-text screening.

Two reviewers (FC and HT or TT or CN) then performed blinded full-text screening against the full eligibility criteria, selecting papers that discussed online videos on open-sourced social media platforms and measured quantitative health outcomes. Disagreement between the 2 reviewers was resolved by consensus and a third reviewer when necessary.

### Study Outcomes

The primary outcome of the systematic review was the impact of the health-related video intervention on quantitative health–related outcomes as specified by each paper.

Additional outcomes included the following: study participant characteristics and sample size; video intervention characteristics, including the method of intervention delivery to the participants, social media platform used, video length, video content, and the use of any theory or frameworks in its development; and the quantitative measure of change in the health-related outcome.

### Data Extraction

Data extraction took place on the Covidence platform. Two reviewers (FC and HT or TT) performed the full data extraction process in a blinded and independent manner. Disagreement was resolved by consensus and a third reviewer when necessary.

Data collection followed the study outcomes as described above. In addition, the following data were collected: study information, study setting, study design, protocol registration, ethical approval status, funding sources, conflict of interest, and statistical analysis methods.

### Risk of Bias Assessment

As it was expected that the review would include studies of different designs, appropriate tools were selected to assess the risk of bias according to the anticipated methodologies used by the studies. For RCTs, the Cochrane Risk of Bias 2 was used [35]. For nonrandomized studies, the ROBIN-I tool was used [36]. For cross-sectional studies, the National Institutes of Health Quality Assessment Tool for Observational Cohort and Cross-Sectional studies was considered more appropriate given it is specifically tailored for observational studies; therefore, selected [37]. This was in addition to the methods described in the registered protocol.

Two reviewers (FC and HT or TT) independently evaluated the risk of bias. Disagreement was resolved by consensus and a third reviewer when necessary.

### Narrative Synthesis

Given the anticipated wide range of health conditions and outcomes encompassed within this search, data were synthesized using a narrative synthesis approach according to the study outcomes: the participants described in the study, the health condition of interest, the video intervention, the methods of delivering the video to the participants, video characteristics, the comparator, the outcome measures of interest, and the effect of the video on the specific outcomes.

### Quantitative Synthesis

The included studies were evaluated for their suitability for meta-analysis. If 2 or more studies were sufficiently homogeneous in terms of participants, interventions, and health outcome measures to provide a meaningful summary, meta-analysis would be performed. The DerSimonian and Laird random effects models with the inverse variance method were used to generate the summary measures of effect in the form of standard mean difference (SMD) to account for similar outcomes measured using different assessment tools [38,39]. SMD was calculated using change from baseline or point measure mean values and SDs for intervention and control groups for each study with relevant outcome data [39]. For studies that did not report SD of changes from baseline, this was imputed from baseline and final SDs using the standard methods described, assuming the correlation coefficient of 0.5 [39]. When necessary, mean values were standardized to reflect the direction of the scale [39]. For study outcomes derived from multiple outcome measures, the most reported measure across studies was selected. If there was no clear indication of the relative importance of the measures, these were combined into a single effect size by averaging the SMDs and calculating the variance, accounting for the correlation between outcome, assuming a correlation coefficient of 0.5 [39]. The resulting composite SMD and variance were used in the subgroup meta-analysis. Statistical heterogeneity would be examined using the standard methods described [38,39]. The *I*^2^ was used to quantify the magnitude of statistical heterogeneity between studies, where *I*^2^ of 30% to 60% represents moderate and *I*^2^ of >60% represents substantial heterogeneity [38]. A meta-analysis would be performed using the R *meta* package [40].

For all interventional studies, an albatross plot was constructed to allow the *P* values to be interpreted in the context of study sample size. The contour lines of the albatross plots were formed by hypothetical effect sizes [41]. *P* values were calculated from the SMD values using the Wald test [42]. In addition, different colors were used to facilitate the visualization of outcomes by subgroup. The albatross plot was made using the R *metap* package [43].

## Results

### Study Selection

**Figure 1.**
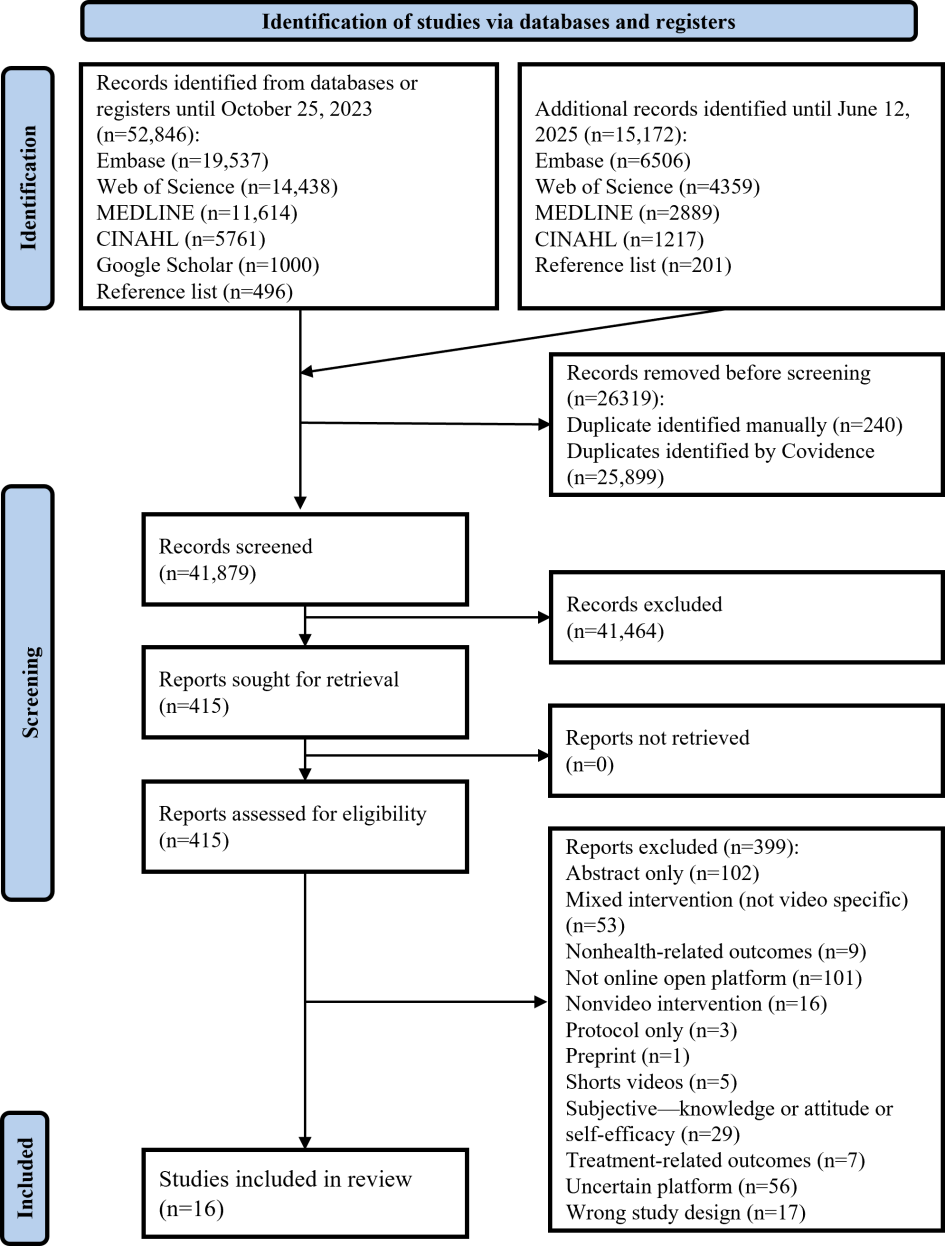
PRISMA (Preferred Reporting Items for Systematic Reviews and Meta-Analyses) flowchart.

A systematic search was conducted on October 25, 2023, and was updated on June 12, 2025, yielding a total of 41,182 records after duplicate removal. After abstract and title screening, 415 papers were included for full-text review, leading to 15 studies being identified as suitable for inclusion [44-58]. In addition, 1 study was added after screening the reference list of the included studies [59]. The PRISMA flow diagram is shown in Figure 1.

### Study Characteristics

The characteristics of the 16 included studies are shown in MultimediaAppendices 2  3. There were 10 RCTs [44-52,59], one of which was of a cross-over design [52], 1 quasi-experimental study [53], 2 pre-post studies [54,56], and 3 observational studies [55,57,58]. Seven studies delivered interventions within health care facilities [44,47,51,53-55,59], 5 in educational institutions [45,49,50,52,57], and 4 online [46,48,56,58].

### Participant Characteristics

Two studies were performed in children [47,59], 1 in children and their parents [51], 5 in adolescents and young adults [45,46,49,50,57], and the remaining in the adult population 18 years or over [44,48,52-56,58].

Overall, 4158 participants were recruited across studies, of which 4097 were analyzed after taking into account participant dropouts. The number of recruits per study ranged from 32 [52] to 1160 participants [57]. The mean age of the participants was 27.1 years (excluding studies that presented age in median only [48] and those that did not present age data [58]). Female participants accounted for 66.7% (n=2734) of the participants across the studies. Detailed participant characteristics are presented in MultimediaAppendices 2  4.

### Health Conditions

Ten (63%) out of the 16 studies assessed the effectiveness of an online video intervention on a mental health–related condition [44-47,52,54-56,58,59]. Among these, 1 study assessed the impact of videos on health care workers’ mental health relating to the COVID-19 pandemic [44]. Four studies used social media videos to alleviate peri-procedural anxiety [47,54,55,59], including 3 that assessed the impact of watching selected videos during the preoperative period on preoperative anxiety [54,55,59]; 1 study examined the efficacy of videos in reducing anxiety relating to dental procedures [47].

Two studies used video interventions to improve participants’ lifestyles; 2 studies examined the effects of YouTube video–delivered physical activity intervention on young adults’ physical activity [49,50]; and 1 study investigated the impact of watching food videos on social media on food consumption, appetite, and BMI [57].

Other health conditions targeted by video interventions included pain relating to knee osteoarthritis [49], patients with coronary artery disease undergoing cardiac catheterization [54], and fear of topical steroid treatment [51].

### Video Intervention

#### Delivery of the Video Intervention to Participants

##### Overview

All videos were hosted on publicly available social media platforms, meaning that any individual could access and view the video content online, unlimited to study participants. Twelve studies hosted videos on YouTube [44-47,49,50,52,54-56,58,59]. All except for 3 studies intentionally delivered the videos to a predetermined group of participants and evaluated their health impact on this group [44-54,56,59]. The following methods of delivery to study participants were described in the included studies.

##### Delivered Through Weblinks

Three studies provided participants with the weblink of the specified social media videos and instructed participants to watch the videos at specific time points [44,50,51]. In 1 study, patients were given a link to the social media website, and they could search the video using keywords such that they could watch it asynchronously at their convenience [53].

##### Delivered Directly to Participants

In 5 studies, participants were directly shown the video sequence in a controlled environment, including the following ways: shown on large screens in the preoperative waiting room [59], shown prior to dental procedures [47], shown through personal cell phones in a testing room [52], or shown to participants in a clear area of a quiet classroom at a school [45]. In 1 study, patients were shown the video through a variety of tools prior to their surgery, including digital monitor devices connected to a hospital smart bed system, or through any devices with internet access by entering the video URL or with QR code scanning [53].

##### Embedded Within Surveys

In 3 studies, video interventions were embedded within a randomized or single-arm pre-post online survey [46,48,56], of which 2 studies used the Qualtrics platform [48,56] and 1 used the Social Science Survey platform [46]. Participants were invited to complete the survey through audience-specific social media platforms [46,56], invitation flyers distributed around university departments [46], invitation shown in the YouTube description section of the intervention video [56], and through a consumer network for digital survey–based research [56].

##### Embedded Within YouTube Channels

In a 2-arm RCT investigating the impact of social media videos on young individuals’ physical activity, participants were instructed to subscribe to the YouTube channel for their respective study groups [49]. They would watch 1 video per week that was uploaded onto each channel, and they were instructed not to consume other YouTube videos relating to physical activity during this time period [49].

##### Observational Studies

Three studies were observational rather than interventional. One prospective study compared the preoperative anxiety of individuals who have watched operation-related videos against those who have not watched such videos within 1 week prior to the operation [55]. The remaining 2 were cross-sectional studies. Tazeoğlu et al [57] investigated the association between self-reported watching of social media food videos and individuals’ weight and BMI. Participants were asked to recall the frequency in which they had watched any food videos on social media platforms. Shin et al [58] uploaded a cross-sectional survey onto a specific YouTube channel that produces sleep-aid related content and invited the channel’s audience to report how watching videos on the channel has impacted their quality of sleep.

### Video Content

Four studies used videos to guide participants through an activity, including Yoga Nidra [44], workout routines [45,49,50], and autonomous sensory meridian response sound [56]. Seven studies placed greater focus on information provision in educating participants about procedures [47,53-55] or on specific health conditions including osteoarthritis [48], suicide prevention in depression [46], and atopic dermatitis and its treatment [51]. The remaining studies offered participants greater flexibility in selecting the videos to watch. One study investigating preoperative anxiety selected videos from a list of age-appropriate video clips based on participants’ individual preference [59]. In Oppenheimer et al [52], participants could watch any videos from a YouTube playlist of 8 nonevocative videos. In 2 studies, participants watched any videos on specified topics (videos on how to perform an impacted tooth extraction [55] and food-related videos [57]) or videos on a specific YouTube channel providing sleep aid [58].

Three studies mentioned the use of theories and/or frameworks in developing videos [48,49,51], including the following: constructivism, social-cognitive theory and information-motivation-behavioral skills model [48], social determination theory [49], storytelling, and behavior change technique taxonomy version 1 [51]. Notably, the videos in these 3 studies were evaluated with their audience prior to their formal delivery to participants. Videos were evaluated through consumer panels [48]; survey and focus groups [49]; and a panel of patients, family members, and health care professionals [51].

### Video Format

Of the studies that reported video lengths, they ranged from 1 minute 25 seconds to 30 minutes [44,52]. Six out of the 16 studies used 1 video only [44,46,48,53,54,56], while the remaining incorporated several videos as part of the intervention [45,47,49-52,55,57-59]. Participants were either shown the video at a single time point [46-48,52,54,56,59] or at regular time intervals [44,45,49,50] or at the participant’s time of convenience [51,53,55,58].

### Risk of Bias

Among the RCTs, 1 study showed low risk of bias [49], while the remaining studies showed some concern [46,47,51,59] or high risk of bias [44,45,48,50,52]. Of the four nonrandomized controlled studies, all have shown moderate [53,54,58], serious [55,57], or critical [56] risk of bias. For the RCTs, the most reported bias was in the selection of the reported results, whereas for the non-RCTs, it was the bias in the measurement of outcomes. The summarized risk of bias assessment of the included studies is shown in Multimedia Appendix 5 for RCTs [43-51,58], and non-RCTs [52-55], and Multimedia Appendix 6 for the full assessment.

### Assessing the Impact of Online Videos

The primary outcome of the systematic review was the impact of the health-related video intervention on quantitative health–related outcomes as specified by each paper.

#### Narrative Synthesis

Two RCTs [47,59] and 1 pre-post [54] study all demonstrated that online video interventions can significantly improve peri-procedural anxiety including perioperative anxiety and dental anxiety (*P*≤.001). One prospective cohort study, however, reported that watching social media videos on tooth extraction 1 week prior to the procedure may be associated with a greater level of anxiety (*P*<.05) [55]. While 2 studies measured procedural-related anxiety using self-reported measures [54,55], 2 assessed outcomes by independent assessors [47,59], and 1 RCT measured 1 component of preprocedural anxiety using heart rate measured with a finger pulse oximeter [47].

In terms of other mental health–related outcomes, 1 study demonstrated that an online video depicting personal stories of how to cope with depression significantly alleviated suicidal ideation (*P*=.04) [46], and a video showing Yoga Nidra to health care workers during COVID-19 duty period improved insomnia (*P*=.02) [44]. One study that showed videos that triggered autonomous sensory meridian response in participants led to improved mood (*P*=.002) and lower levels of arousal (*P*<.001) [56]. No significant impact was demonstrated on participants’ levels of depression [44] or stress [45]. One crossover RCT comparing watching a nonevocative YouTube playlist against browsing social media platforms (Facebook and Instagram) showed that both interventions reduced the level of stress, and there was no significant difference between the 2 interventions [52]. Most studies describing mental health–related outcomes were measured using self-completed questionnaires; 1 study measured the level of stress objectively using arm-band continuous heart rate monitoring and individual pre- and postintervention cortisol levels [52].

Two studies assessed the impact of videos on self-reported quality-of-life outcomes: an RCT of online videos that described atopic dermatitis and its treatment, and 1 quasi-experimental study of showing a video prior to cardiac catheterization. No significant impact was found in either study, although watching online videos prior to cardiac catheterization improved spiritual well-being (*P*<.001) [51,53].

One RCT with low risk of bias found that a video grounded in self-determination theory significantly improved young adults’ level of physical activity compared with watching a general health video [49]. The study measured the level of physical activity and sleep quality of its participants using a wrist-worn ActiGraph Link GT9X accelerometer [49]. Another RCT examining the impact of a video intervention on executive function, measured using self-completed tasks by participants, did not demonstrate significance [45].

Two RCTs that incorporated appropriate theories and/or frameworks in developing videos showed a significant impact on improving patients’ self-sufficiency in managing pain relating to knee osteoarthritis [48], and in reducing parents’ fear of topical corticosteroid, respectively, although the latter study did not have a significant impact upon disease severity or the family’s quality of life [51].

Of the two cross-sectional studies, 1 study demonstrated a positive correlation between those who regularly watch food videos and individuals’ BMI, while the other reported effectiveness in YouTube-delivered mind-body interventions in improving sleep quality [57,58].

Nine studies used solely self-reported outcome measures [46,48,50,53-56,58]. Five studies incorporated predominantly objectively measured outcomes or observer-determined outcomes [47,49,52,57,59].

#### Quantitative Synthesis

**Figure 2.**
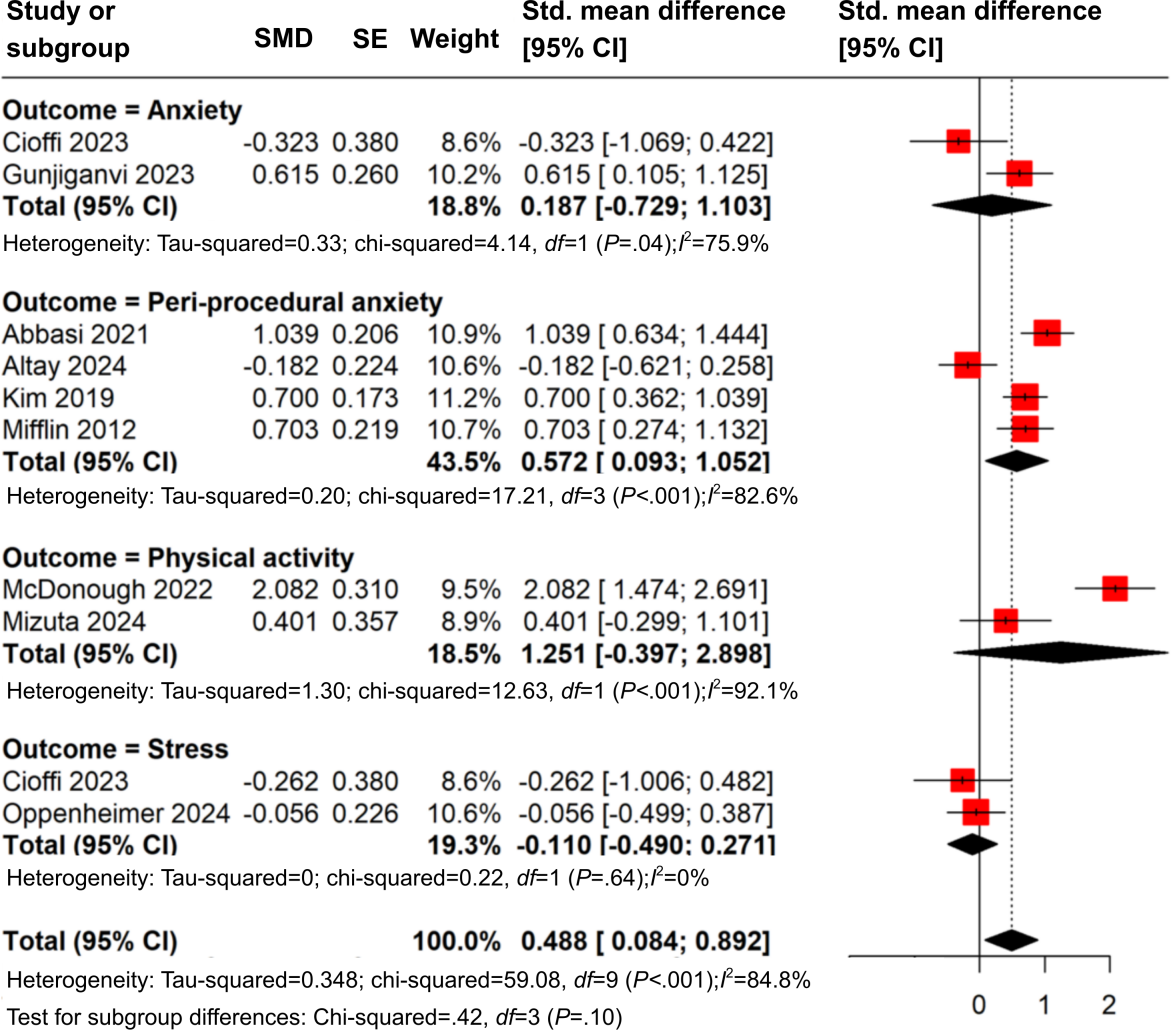
Forest plot of standard mean differences (SMDs) for intervention effects across outcomes. Pooled estimates were calculated using random-effects models. Diamonds represent the overall effect size for each outcome and the total pooled effect size. Horizontal lines indicated 95% CI. Study weights are shown as percentages. Heterogeneity was assessed using the *I*^2^ statistic and Tau^2^ [44,45,47,49,50,52,54,55,59].

The primary outcomes and measures of the included studies were categorized based on outcome types, and the numerical values, calculated standard mean difference, and 2-tailed *P* values in the context of the studies’ risk of bias are shown in Multimedia Appendix 4. While the included studies displayed variations in terms of study design, participants, and the range of health outcomes, a subgroup meta-analysis was conducted to examine the impact of online video interventions on different health-related outcomes, including anxiety, peri-procedural anxiety, physical activity, and stress (Figure 2) [45,47,49,50,52,54,55,59]. Stratified by outcomes, for peri-procedural anxiety, a significant moderate effect was observed (SMD=0.57, 95% CI 0.09‐1.05, *I*^2^ 82.6%), although studies were heterogeneous in terms of study design (RCTs and non-RCTs) and participant characteristics (both adults and children). For physical activity, a large yet nonsignificant effect was identified (SMD=1.25, 95% CI -0.40‐2.90, *I*^2^ 92.1%), and the 2 included studies were both RCTs with participants of similar age. No significant effects were identified for anxiety and stress. Notably, all but 1 study included in sub-group meta-analysis displayed moderate-to-high risk of bias.

**Figure 3.**
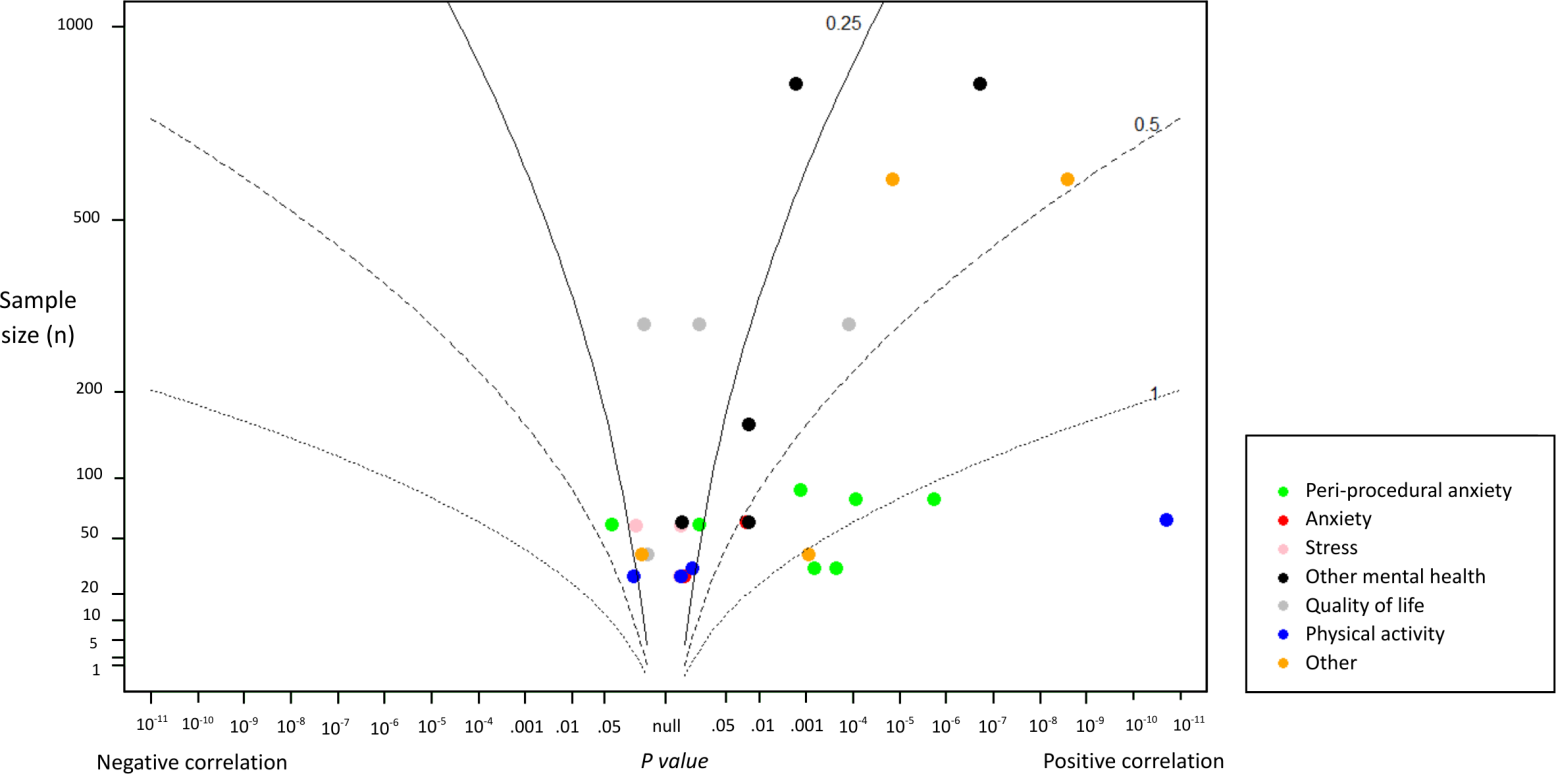
Albatross plot of *P* values against study sample size. The plot was constructed to visually summarize the distribution of study results by mapping *P* values against sample sizes (**N**). The vertical axis represents sample size on a logarithmic scale (ranging from 1 to 1000), while the horizontal axis displays *P* values on a 2-sided scale, with negative correlations plotted to the left and positive correlations to the right. Contour lines indicate approximate effect sizes expressed as standardized mean differences (SMD=0.25, 0.5, 1.0). Each point corresponds to a particular outcome of an individual study, color-coded according to outcome domain (eg, peri-procedural anxiety, stress, quality of life, physical activity). Studies falling closer to the center line suggested weaker or null effects, whereas those positioned further toward the contour boundaries indicate stronger associations.

An albatross plot (Figure 3) was used to visualize the primary outcomes across all interventional studies. Most studies were of sample sizes between 50 and 200. A concentration of points could be observed within the small-to-moderate effect size contours (SMD 0.25‐0.50). Notably, peri-procedural anxiety appeared to be more significantly associated with watching social media videos across studies, while other outcomes appeared more dispersed. One RCT on the effect of a behavioral framework–informed video on physical activity displayed the greatest effect size among all studies [49].

## Discussion

### Principal Results

To our knowledge, this is the first systematic review to comprehensively evaluate the impact of social media videos on quantitative health outcomes. The evidence indicates that social media videos can positively influence health outcomes, particularly by reducing peri-procedural anxiety. When interventions were developed with behavioral or psychological theories and were informed by audience input, they demonstrated stronger and more consistent improvements in study-specific health outcomes.

Most studies utilized self-reported outcome measures, and significant gaps persist in measuring the impact of online videos on physical health–related outcomes. Of the studies that measured physical health–related outcomes, including disease severity and 90-day readmission rate, no significant impact was found [50,51], with the exception of McDonough et al [49], which demonstrated that a theory-based video, tailored to audience preferences, significantly improved physical activity levels.

Notably, all but 1 study demonstrated moderate-to-high risk of bias [49].

### Comparison With Prior Work

The most consistent finding across the included studies was the positive impact of online videos in reducing peri-procedural anxiety. This was aligned with a previous systematic review on video-based preoperative patient education—not specific to social media videos—in which the majority of the studies (six out of eight) reported that preoperative videos significantly lowered patients’ anxiety scores [60]. The preprocedural period can often be psychologically burdensome for patients. Preparatory information shown visually through videos can potentially help to alleviate anxiety by clarifying what to expect during medical procedures. Interestingly, videos tailored to patients’ preferences were as effective in reducing anxiety as those explaining the medical procedures themselves, suggesting mechanisms beyond patient education. Nevertheless, when examining the impact of videos on peri-procedural physical and quality-of-life outcomes, the findings of this systematic review were consistent with previous studies that no significant changes were demonstrated [53,54,60]. This may suggest that a video intervention provided preoperatively may not lead to enough change in individuals’ knowledge, attitude, and behaviors, such that individuals’ postoperative recovery can be impacted, which can lead to improved physical outcomes.

Several studies in our review described the use of online video as a medium to deliver specific interventions to improve individuals’ health outcomes. For instance, Gunjiganvi et al described the use of Yoga Nidra, a form of meditative procedure described by its practitioners to induce a calm inner stillness [44,61]. Previous studies examining the effectiveness of in-person Yoga Nidra, including RCTs, have similarly demonstrated its effectiveness in alleviating anxiety, depression, and insomnia [62-64], as well as physical outcomes such as blood pressure and lipid profiles [65,66]. It appeared that the delivery of Yoga Nidra through the video format can result in similar improvement in the participants’ mental health–related outcomes [44]. Similarly, when participants were instructed to follow a series of physical activity videos, described in McDonough et al [49], the level of physical activity of the participants was significantly increased. Nevertheless, it remains uncertain as to whether such improvement persisted postintervention.

Regarding video content, 3 studies developed videos incorporating theories or frameworks, and with input from their target audience [48,49,51]. All have demonstrated significant positive impact on their respective outcomes, including psychological-cognitive outcomes in people with knee pain, levels of physical activity, and fear of topical steroid treatment [48,49,51]. Such findings align with a previous systematic review assessing the effectiveness of video-based intervention in health promotion, which highlighted the importance of incorporating theoretical frameworks to guide message development in future video interventions [67].

### Limitations

Our systematic review has several limitations. First, we restricted our search to databases containing peer-reviewed papers and excluded studies lacking full methodological details, such as meeting abstracts and proceedings, to ensure the quality and completeness of our review. However, this may have excluded relevant methodologies and outcomes discussed in the broader literature. Second, to ensure broad coverage of health-related conditions, we used wide-ranging search terms, resulting in a large number of papers for screening. Given resource constraints, this systematic review was limited by having 1 reviewer for the abstract and title screening, which could increase its vulnerability to selection bias. To reduce such bias, papers with unclear eligibility were conservatively included, and the full text screening phase involved 2 reviewers to ensure concordance. Third, we focused exclusively on long-form social media videos, as audience interaction with “Shorts” videos can vary substantially. “Shorts”—brief, algorithmically surfaced video clips designed for quick consumption—have grown in popularity in recent years, reshaping how audiences engage with content, but such format has been associated with the risk of online addiction [28,29]. The findings from this systematic review should thus be generalized to long-form social media videos.

Importantly, all except for 1 included study have displayed moderate-to-high risk of bias, and the most reported bias stemmed from the measurement and reporting of study outcomes [49]. Such findings reemphasized the importance of utilizing more objective methods in tracking health-related outcomes beyond self-reported tools. While the systematic review reported a significant positive impact of social media videos on peri-procedural anxiety, the 4 included studies all showed a moderate-to-high risk of bias and are heterogeneous in terms of study designs (both RCT and non-RCTs) and participant demographics (children and adults); therefore, future studies should validate such findings with better designed studies, more robust form of measuring the outcomes beyond self-reported tools, and in a specified population.

### Future Work and Implications

Social media is characterized by its bidirectional, interactive nature, and previous research has shown the paramount importance of engaging community partners when designing public health messaging in order to build trust and ensure its effectiveness [25,68]. Nevertheless, all but 2 studies in this systematic review delivered the videos in a unidirectional manner to a predetermined group of participants in a controlled, experimental setting, without clear opportunities for reciprocal interactions. Two studies attempted to better simulate how individuals typically interact on social media platforms: 1 utilized features on the YouTube platform by creating 2 separate channels for participants in experimental and control groups, yet the audience was preselected with no clear monitoring of their online interactions [49]; 1 cross-sectional study directly posted the online questionnaire onto the YouTube channel containing sleep-aid video interventions, allowing respondents to better reflect how users typically behave on such platforms [58].

The benefit of interactivity on social media has been highlighted in previous studies among individuals suffering from mental illnesses [5,69]. Individuals find ease of connecting with each other anonymously for mutual support, especially when they may have few social contacts offline and may suffer from highly stigmatizing conditions [5,69]. On the other hand, the negative impact of social media cannot be undermined, including the potential for mass misinformation and disinformation, and its link with online addiction, associated with the platforms’ algorithmic nature [70-72]. Future studies should focus on assessing the health impact of social media videos incorporating the full range of functionalities native to the platform, such as likes, shares, and comments on the YouTube platform, as well as the platform’s algorithmic nature, by which users receive individualized content that reflects their viewing history [73]. While the included studies of this systematic review tended to show 1 or a predetermined series of videos, the users of such platforms may view a series of videos as determined by the algorithm. How such features can positively or negatively impact upon their users’ health outcomes is important to be explored, especially in evaluating how social media can impact upon the traditional health care services, and whether, or indeed, how such interventions can be incorporated into the health care workflow.

This systematic review found that the assessment of the health impact of social media videos primarily focused on mental health-related or more subjectively reported outcomes, such as pain and fear. Previous systematic reviews of health-related video interventions have described their use for a broader range of purposes in health care, including health promotion such as disease prevention (eg, nutrition, vaccination), detection (eg, cancer screening and self-examination), and prevention of risky behaviors (eg, smoking, binge drinking) [67], as well as hospital-based education to change outcomes relating to heart disease, cancer, stroke, sleep apnea, and diabetes [74]. Several studies in this systematic review have attempted to measure objective physical health–related outcomes, such as the use of heart rate and physical activity trackers, and the measure of cortisol levels [49,52]. Nevertheless, given the wide-reaching potential of social media to a global audience, our findings highlighted the need to assess a wider range of health outcomes, notably those relating to physical health, utilizing methodological innovations such as the use of digital tracing, passive sensors, and laboratory investigations. Given the existing interactive features on social media platforms, means to directly measure health-related outcomes or their surrogate measures on the platform should be explored, such as the embedding of validated survey tools and digital tracking of user behaviors.

The variable effectiveness of video interventions identified in this systematic review can be influenced by several factors, including the duration and frequency of exposure to intervention, the content and quality of the videos, participant compliance, and whether behavioral theories or frameworks were applied in developing the intervention. As a visual-audio tool, it is necessary to understand the mechanisms that lead to a change in individuals’ health-related outcomes, including the effect of the videos on individuals’ health-related behaviors and their determinants such as knowledge and attitudes. Mental health–related outcomes may be more easily affected by a video, while a change in physical health–related outcomes may require more consistent and interactive interventions. Nevertheless, in 1 RCT of low risk of bias, where participants can directly follow the physical activity videos that were grounded in a behavioral theory, which were consistently delivered to participants on a weekly basis, there was a significant increase in the participants’ physical activity levels [49]. Health care providers and policymakers should thus design future studies to examine how best to deliver social media videos as behavioral interventions, with in-depth behavioral analysis of the individuals affected by the health conditions, so that more targeted video interventions that are grounded in behavioral theories or frameworks can be developed.

### Conclusions

In conclusion, this systematic review has demonstrated a potential positive impact of online social media videos in improving peri-procedural anxiety and the merit of incorporating behavioral theories or frameworks in developing the interventions. Nevertheless, studies have demonstrated a moderate-to-high risk of bias and high heterogeneity in terms of study design, participant demographics, and the range of health conditions. Future studies should focus on the measurement of more objective physical outcomes and the evaluation of video interventions in the context of the interactive and algorithmic features of social media platforms. Health care providers and policymakers should endeavor to incorporate in-depth behavioral analysis of the target populations so as to develop interventions grounded in behavioral theories or frameworks.

## Supplementary material

10.2196/77578 Multimedia Appendix 1 Search strategy.

10.2196/77578 Multimedia Appendix 2 Summary table of the included studies and study core characteristics.

10.2196/77578 Multimedia Appendix 3 Detailed extraction information of all included studies.

10.2196/77578 Multimedia Appendix 4 Study numerical outcomes by subgroups in the context of risk of bias.

10.2196/77578 Multimedia Appendix 5 Risk of bias of the included randomized controlled trials and nonrandomized controlled trials (except for cross-sectional studies).

10.2196/77578 Multimedia Appendix 6 Risk of bias assessment by items for all included studies.

10.2196/77578 Checklist 1 PRISMA checklist.

## Abbreviations

PICO: population, intervention, comparison, outcome
PRISMA: Preferred Reporting Items for Systematic reviews and Meta-Analyses
RCT: randomized controlled trial
SMD: standard mean difference

## References

[1] Social media. Cambridge Dictionary.

[2] Grajales III FJ, Sheps S, Ho K, Novak-Lauscher H, Eysenbach G (2014). Social media: a review and tutorial of applications in medicine and health care. J Med Internet Res.

[3] Tsao SF, Chen H, Tisseverasinghe T, Yang Y, Li L, Butt ZA (2021). What social media told us in the time of COVID-19: a scoping review. Lancet Digit Health.

[4] Braun LA, Zomorodbakhsch B, Keinki C, Huebner J (2019). Information needs, communication and usage of social media by cancer patients and their relatives. J Cancer Res Clin Oncol.

[5] Naslund JA, Bondre A, Torous J, Aschbrenner KA (2020). Social media and mental health: benefits, risks, and opportunities for research and practice. J Technol Behav Sci.

[6] How YouTube search works. YouTube.

[7] Mayer RE (2008). Applying the science of learning: evidence-based principles for the design of multimedia instruction. Am Psychol.

[8] Rolfe VE, Gray D (2011). Are multimedia resources effective in life science education? A meta-analysis. Biosci Educ.

[9] Kesten JM, Bhattacharya A, Ashiru-Oredope D, Gobin M, Audrey S (2017). The Antibiotic Guardian campaign: a qualitative evaluation of an online pledge-based system focused on making better use of antibiotics. BMC Public Health.

[10] Noetel M, Griffith S, Delaney O (2021). Video improves learning in higher education: a systematic review. Rev Educ Res.

[11] Schneider S, Nebel S, Beege M, Rey GD (2018). The autonomy-enhancing effects of choice on cognitive load, motivation and learning with digital media. Learn Instr.

[12] Kopf S, Wilk S, Effelsberg W Bringing videos to social media.

[13] Al-Quran MWM (2022). Traditional media versus social media: challenges and opportunities. Technium.

[14] (2022). 4 stories of living with multiple sclerosis - #mymsmoment. National MS Society YouTube page.

[15] (2022). How can the world solve the climate and biodiversity crises?. Grantham Institute, Imperial College London YouTube page.

[16] (2017). Antibiotic resistance advert—keep antibiotics working and take your doctor’s advice. Chorley and South Ribble and Greater Preston CCG YouTube page.

[17] Bhattacharya A, Hopkins S, Sallis A, Budd EL, Ashiru-Oredope D (2017). A process evaluation of the UK-wide Antibiotic Guardian campaign: developing engagement on antimicrobial resistance. J Public Health (Oxf).

[18] (2025). Health outcomes. Taylor and Francis.

[19] Oleske DM, Islam SS, Doan T, Renz C, Bhattacharya M, Lievano F, Scarazzini L (2019). Pharmacovigilance: A Practical Approach.

[20] Pantaleon L (2019). Why measuring outcomes is important in health care. J Vet Intern Med.

[21] Murray E, Hekler EB, Andersson G (2016). Evaluating digital health interventions: key questions and approaches. Am J Prev Med.

[22] Tuong W, Larsen ER, Armstrong AW (2014). Videos to influence: a systematic review of effectiveness of video-based education in modifying health behaviors. J Behav Med.

[23] Chen J, Wang Y (2021). Social media use for health purposes: systematic review. J Med Internet Res.

[24] Claflin SB, Klekociuk S, Fair H (2022). Assessing the impact of online health education interventions from 2010-2020: a systematic review of the evidence. Am J Health Promot.

[25] Ghahramani A, de Courten M, Prokofieva M (2022). The potential of social media in health promotion beyond creating awareness: an integrative review. BMC Public Health.

[26] Page MJ, McKenzie JE, Bossuyt PM (2021). The PRISMA 2020 statement: an updated guideline for reporting systematic reviews. BMJ.

[27] Oladipo T Ask Buffer: what is short-form video, and how can you use it?. Buffer.

[28] Qin Y, Omar B, Musetti A (2022). The addiction behavior of short-form video app TikTok: the information quality and system quality perspective. Front Psychol.

[29] Chen Y, Li M, Guo F, Wang X (2023). The effect of short-form video addiction on users’ attention. Behav Inf Technol.

[30] Arevalo-Rodriguez I, Smailagic N, Roqué I Figuls M (2015). Mini-Mental State Examination (MMSE) for the detection of Alzheimer’s disease and other dementias in people with mild cognitive impairment (MCI). Cochrane Database Syst Rev.

[31] Pequeno NPF, Cabral NLDA, Marchioni DM, Lima SCVC, Lyra CDO (2020). Quality of life assessment instruments for adults: a systematic review of population-based studies. Health Qual Life Outcomes.

[32] Google Translate.

[33] Bramer WM (2016). Variation in number of hits for complex searches in Google Scholar. J Med Libr Assoc.

[34] Covidence.

[35] Sterne JAC, Savović J, Page MJ (2019). RoB 2: a revised tool for assessing risk of bias in randomised trials. BMJ.

[36] Sterne JA, Hernán MA, Reeves BC (2016). ROBINS-I: a tool for assessing risk of bias in non-randomised studies of interventions. BMJ.

[37] Ma LL, Wang YY, Yang ZH, Huang D, Weng H, Zeng XT (2020). Methodological quality (risk of bias) assessment tools for primary and secondary medical studies: what are they and which is better?. Mil Med Res.

[38] Higgins JPT, Deeks JJ, Altman DG (2019). Cochrane Handbook for Systematic Reviews of Interventions.

[39] Higgins JPT, Li T, Deeks JJ (2019). Cochrane Handbook for Systematic Reviews of Interventions.

[40] (2025). Meta: general package for meta-analysis. The Comprehensive R Archive Network.

[41] Harrison S, Jones HE, Martin RM, Lewis SJ, Higgins JPT (2017). The albatross plot: a novel graphical tool for presenting results of diversely reported studies in a systematic review. Res Synth Methods.

[42] Mansournia MA, Nazemipour M, Etminan M (2022). P-value, compatibility, and S-value. Global Epidemiology.

[43] (2017). Meta-analysis of significance values. The Comprehensive R Archive Network.

[44] Gunjiganvi M, Rai S, Awale R, Mishra P, Gupta D, Gurjar M (2023). Efficacy of yoga nidra on depression, anxiety, and insomnia in frontline COVID-19 healthcare workers: a pilot randomized controlled trial. Int J Yoga Therap.

[45] Cioffi R, Lubetzky AV (2023). BOXVR versus guided YouTube boxing for stress, anxiety, and cognitive performance in adolescents: a pilot randomized controlled trial. Games Health J.

[46] Niederkrotenthaler T, Till B (2020). Effects of awareness material featuring individuals with experience of depression and suicidal thoughts on an audience with depressive symptoms: randomized controlled trial. J Behav Ther Exp Psychiatry.

[47] Abbasi H, Saqib M, Jouhar R (2021). The efficacy of little lovely dentist, dental song, and tell-show-do techniques in alleviating dental anxiety in paediatric patients: a clinical trial. Biomed Res Int.

[48] Egerton T, Bennell KL, McManus F, Lamb KE, Hinman RS (2022). Comparative effect of two educational videos on self-efficacy and kinesiophobia in people with knee osteoarthritis: an online randomised controlled trial. Osteoarthr Cartil.

[49] McDonough DJ, Helgeson MA, Liu W, Gao Z (2022). Effects of a remote, YouTube-delivered exercise intervention on young adults’ physical activity, sedentary behavior, and sleep during the COVID-19 pandemic: randomized controlled trial. J Sport Health Sci.

[50] Mizuta R, Maeda N, Tashiro T (2024). Effectiveness of metaverse space-based exercise video distribution in young adults: randomized controlled trial. JMIR Mhealth Uhealth.

[51] Brunner C, Schlüer AB, Znoj H (2023). Video-based education with storytelling reduces parents’ fear of topical corticosteroid use in children with atopic dermatitis: a randomized controlled trial (the EduDerm study part II). Adv Skin Wound Care.

[52] Oppenheimer S, Bond L, Smith C (2024). Social media does not elicit a physiological stress response as measured by heart rate and salivary cortisol over 20-minute sessions of cell phone use. PLoS ONE.

[53] Peng JR, Su HC, Lin CP (2021). Role of an e-health intervention in holistic healthcare: a quasiexperiment in patients undergoing cardiac catheterization in Taiwan. J Healthc Eng.

[54] Kim MJ, Oh HK, Lee KC (2019). Effects of an internet-based informational video on preoperative anxiety in patients with colorectal cancer. Ann Surg Treat Res.

[55] Altay B, Kale Ş, Basiry MN, Çoban E (2024). The association of social media videos and patients’ preoperative anxiety. J Oral Maxillofac Surg.

[56] Smejka T, Wiggs L (2022). The effects of autonomous sensory meridian response (ASMR) videos on arousal and mood in adults with and without depression and insomnia. J Affect Disord.

[57] Tazeoğlu A, Kuyulu Bozdogan FB (2022). The effect of watching food videos on social media on increased appetite and food consumption. Nutr Clín Diet Hosp.

[58] Shin JW, Yoo J, Kim H (2025). User experiences and effects of expert-led YouTube mind-body interventions on insomniacs: a survey study. J Korean Med Sci.

[59] Mifflin KA, Hackmann T, Chorney JM (2012). Streamed video clips to reduce anxiety in children during inhaled induction of anesthesia. Anesth Analg.

[60] Tom K, Phang PT (2022). Effectiveness of the video medium to supplement preoperative patient education: a systematic review of the literature. Patient Educ Couns.

[61] Pandi-Perumal SR, Spence DW, Srivastava N (2022). The origin and clinical relevance of yoga nidra. Sleep Vigil.

[62] Moszeik EN, von Oertzen T, Renner KH (2022). Effectiveness of a short yoga nidra meditation on stress, sleep, and well-being in a large and diverse sample. Curr Psychol.

[63] Musto S, Hazard Vallerand A (2023). Exploring the uses of yoga nidra: an integrative review. J Nurs Scholarsh.

[64] Datta K, Tripathi M, Verma M, Masiwal D, Mallick HN (2021). Yoga nidra practice shows improvement in sleep in patients with chronic insomnia: a randomized controlled trial. Natl Med J India.

[65] Ahuja N, Bhardwaj P, Pathania M (2024). Yoga Nidra for hypertension: a systematic review and meta-analysis. J Ayurveda Integr Med.

[66] Anjana K, Archana R, Mukkadan JK (2022). Effect of om chanting and yoga nidra on blood pressure and lipid profile in hypertension—a randomized controlled trial. J Ayurveda Integr Med.

[67] Xiao X, Wong RM, Yang W (2024). Effectiveness of video-based health promotion: a systematic review and meta-analysis. Patient Educ Couns.

[68] de Vere Hunt I, Linos E (2022). Social media for public health: framework for social media-based public health campaigns. J Med Internet Res.

[69] Naslund JA, Grande SW, Aschbrenner KA, Elwyn G (2014). Naturally occurring peer support through social media: the experiences of individuals with severe mental illness using YouTube. PLoS ONE.

[70] Richards D, Caldwell PHY, Go H (2015). Impact of social media on the health of children and young people. J Paediatr Child Health.

[71] Moorhead SA, Hazlett DE, Harrison L, Carroll JK, Irwin A, Hoving C (2013). A new dimension of health care: systematic review of the uses, benefits, and limitations of social media for health communication. J Med Internet Res.

[72] Pellegrino A, Stasi A, Bhatiasevi V (2022). Research trends in social media addiction and problematic social media use: a bibliometric analysis. Front Psychiatry.

[73] Zollo F, Baronchelli A, Betsch C, Delmastro M, Quattrociocchi W (2024). Understanding the complex links between social media and health behaviour. BMJ.

[74] Dahodwala M, Geransar R, Babion J, de Grood J, Sargious P (2018). The impact of the use of video-based educational interventions on patient outcomes in hospital settings: a scoping review. Patient Educ Couns.

