# Supplementary material for: The Impact of Social Media Videos on Quantitative Health Outcomes: Systematic Review
Source: JMIR Infodemiology. 2026 Feb 19;6:e77578. doi: 10.2196/77578 (PMC12919906; doi:10.2196/77578)
Supplement: Multimedia Appendix 1 [file infodemiology-v6-e77578-s001.docx]

**Appendix 1: Search Strategy**

**MEDLINE**

1. Online OR internet OR "social media".mp
2. Exp Internet/
3. 1 OR 2
4. (video* OR animation*).mp
5. Exp video-audio media/
6. 4 OR 5
7. 3 AND 6
8. YouTube OR vimeo OR dailymotion OR "facebook watch".mp
9. 7 OR 8
10. (health* or medic* or patient*).mp
11. 10 AND 9

**EMBASE**

1. (online or internet or (social and media)).mp
2. Exp internet/
3. Exp social media/
4. Social network/
5. 1 OR 2 OR 3 OR 4
6. (video* OR animation*).mp
7. Exp videorecording/
8. 6 OR 7
9. 5 AND 8
10. YouTube or vimeo or dailymotion or "facebook watch"
11. 9 OR 10
12. Health* OR medic* OR patient*
13. 11 AND 12

**Web of Science**

1. ALL=(Online OR internet OR "social media")
2. ALL=(Video* OR animation*)
3. #2 AND #1
4. ALL=(YouTube or vimeo or dailymotion or "facebook watch" )
5. #4 OR #3
6. ALL=(Health* OR medic* OR patient*)
7. #6 AND #5

**CINAHL**

1. Online or internet or “social media”
2. Video* OR animation*
3. S2 and S3
4. YouTube OR vimeo OR dailymotion OR "facebook watch"
5. S4 OR S5
6. Health* OR medic* OR patient*

S6 AND S7
