# Supplementary material for: The Impact of Social Media Videos on Quantitative Health Outcomes: Systematic Review
Source: JMIR Infodemiology. 2026 Feb 19;6:e77578. doi: 10.2196/77578 (PMC12919906; doi:10.2196/77578)
Supplement: Multimedia Appendix 5 [file infodemiology-v6-e77578-s005.docx]

**Appendix 5. Risk of bias of RCTs and non-RCTs. 1). Risk of bias assessment for randomized controlled studies using ROB-2 tool; 2). Risk of bias assessment for non-randomized controlled studies using ROBIN-I tool.**


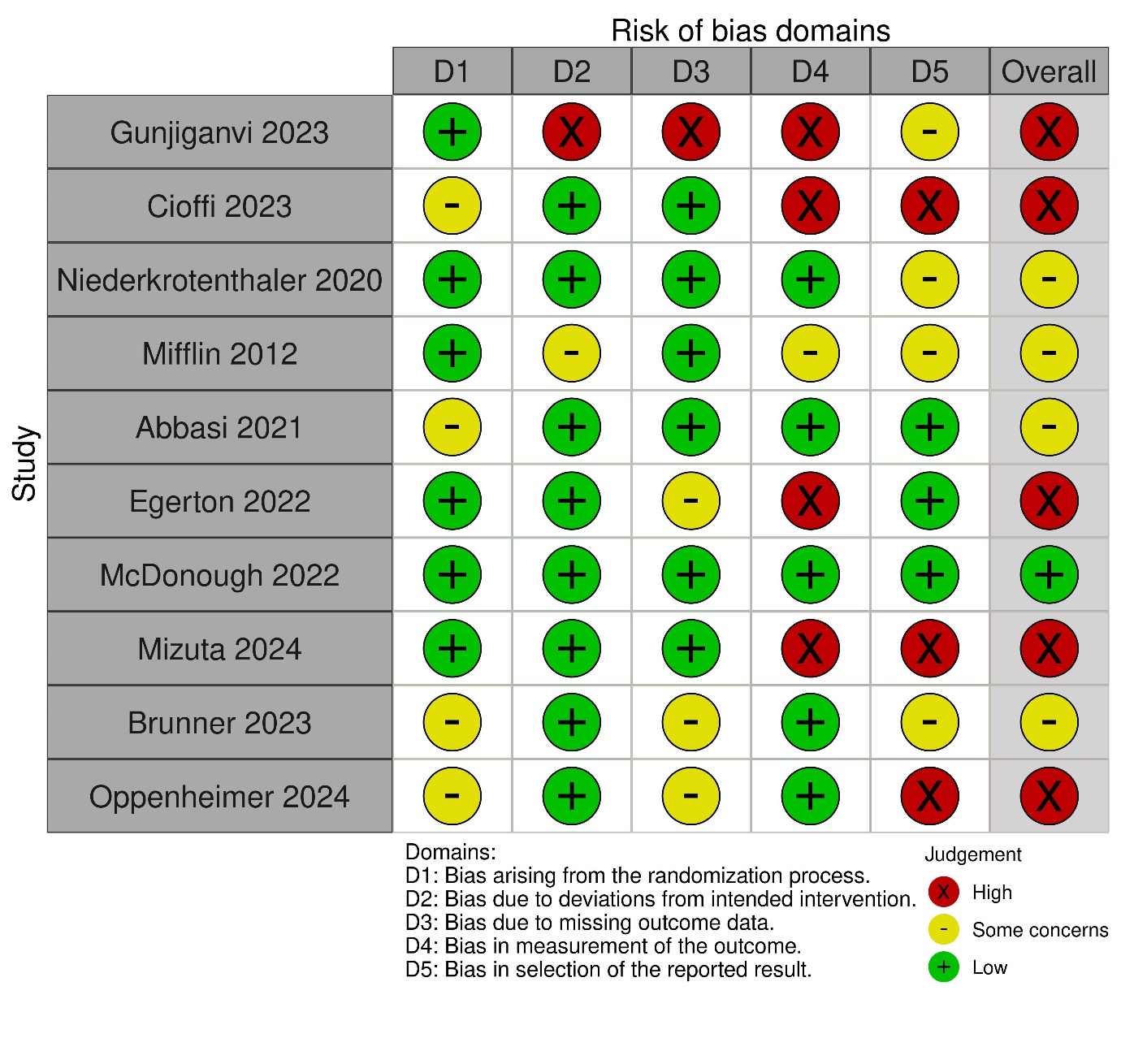

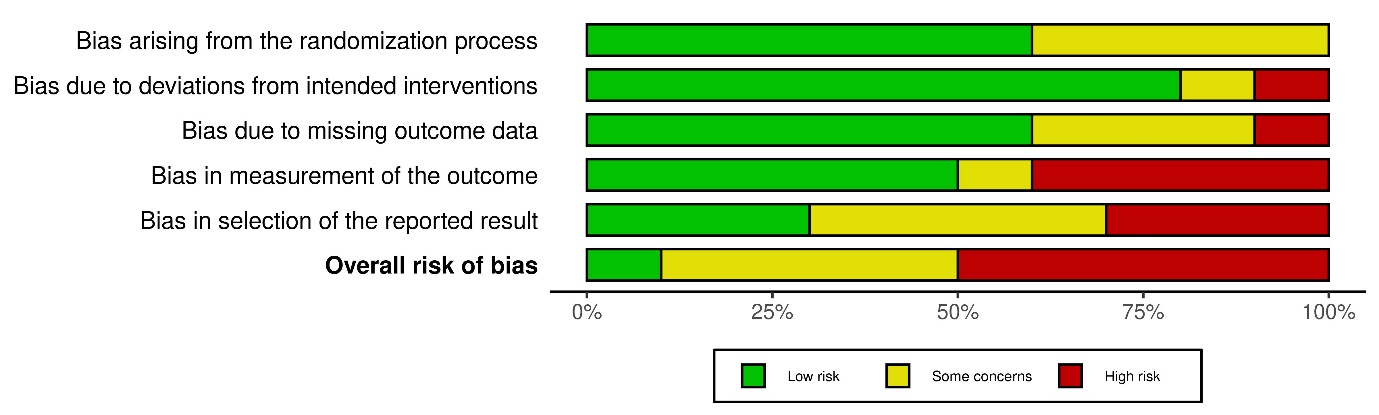


**1).**

**
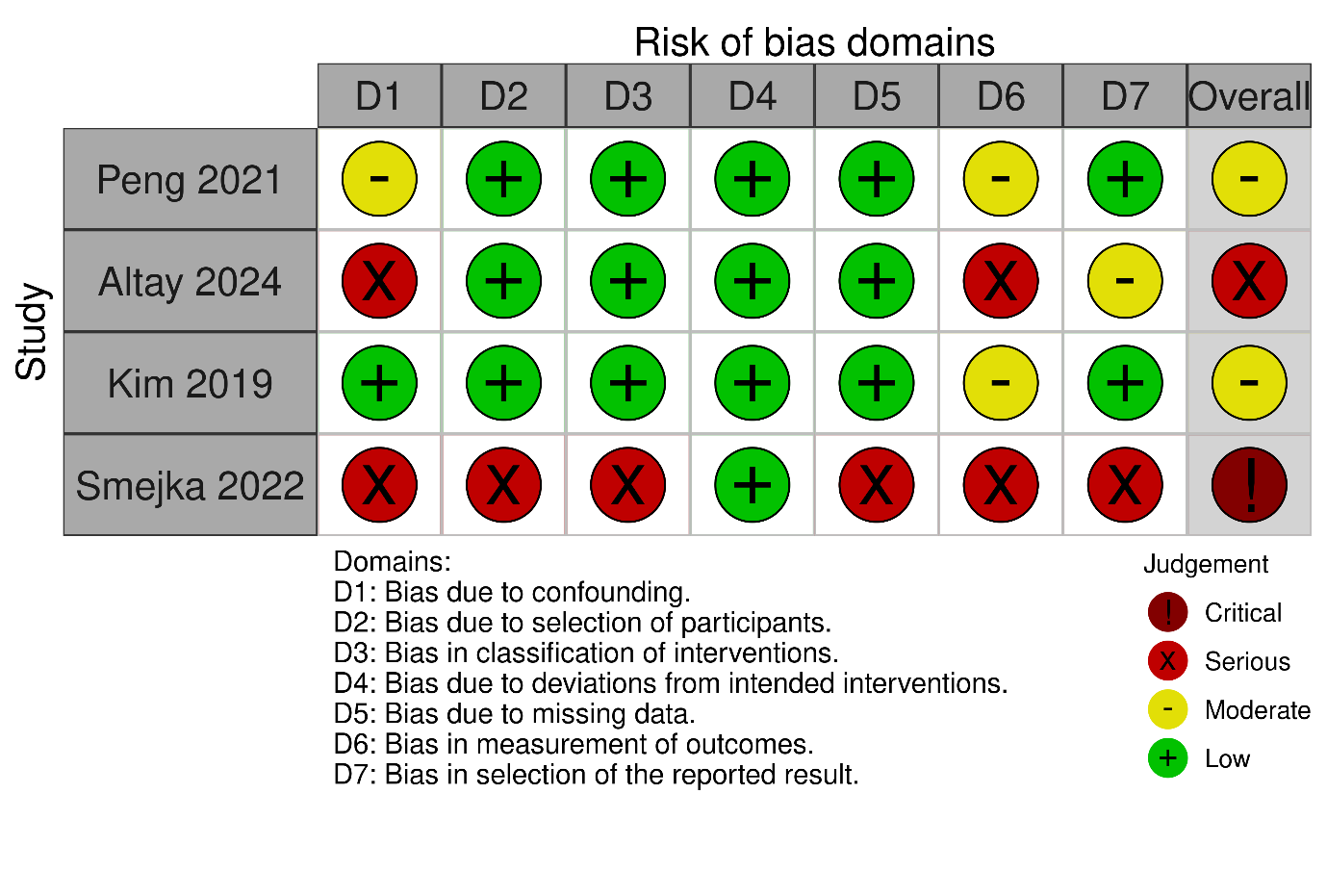

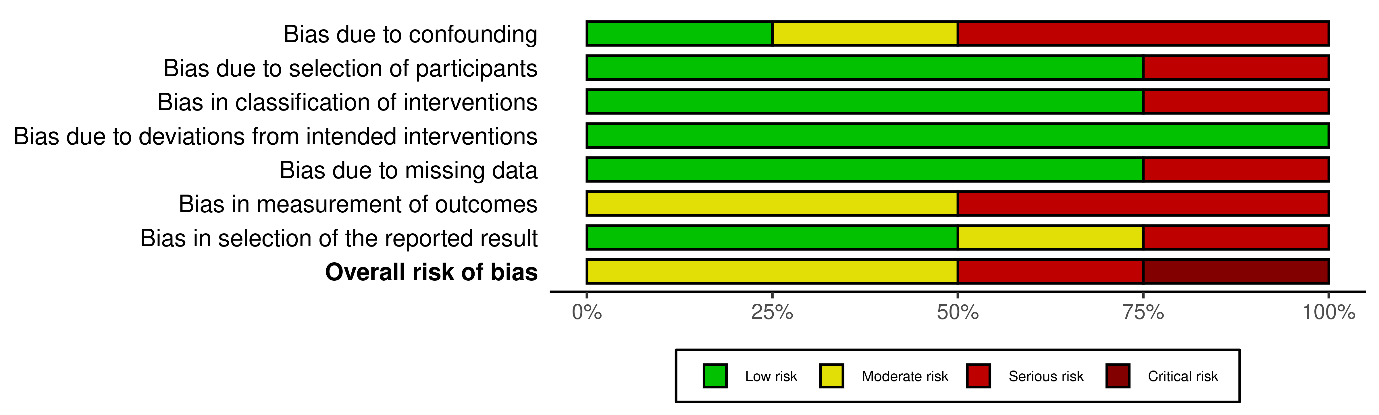
**

**2).**
